# Epidemiology of Acid-Burns in a Major Referral Hospital in Tehran, Iran

**Published:** 2017-05

**Authors:** Reza Vaghardoost, Jafar Kazemzadeh, Mostafa Dahmardehei, Soheila Rabiepoor, Ramyar Farzan, Ali Asghar Kheiri, Rahman Khosravy, Farzad Manafi

**Affiliations:** 1 Department of Plastic Surgery, Iran University of Medical Sciences, Tehran, Iran;; 2 Reconstructive and Burn surgeon, Urmia University of Medical Sciences, Urmia, Iran;; 3 Reproductive Health Research Center, Midwifery Department, Urmia University of Medical Sciences, Urmia, Iran;; 4 Department of Plastic Surgery, Rasht University of Medical Sciences, Gilan, Iran;; 5 Department of Plastic Surgery, Tabriz University of Medical Sciences, Tabriz, Iran;; 6 Department of Surgery, Urmia University of Medical Sciences, Iran

**Keywords:** Burn, Acid, Injury, Epidemiology, Iran

## Abstract

**BACKGROUND:**

Most of the acid- burns are due to assault or accidental. The epidemiology of burns is diverse across the world and within a country. We evaluated the epidemiology and outcome of acid-burns in tertiary health care center in Tehran, Iran.

**METHODS:**

This study was retrospective descriptive among patients referred for acid-burn injury that was done in a referral Burn Care Center in Tehran, Iran, during a ten-year period since 2005 to 2014. Patient’s data collected by a specially designed check list. The subjects included 37 consecutive patients with various causes of acid burn injury. Descriptive statistics (means with standard deviations or frequency distribution) of sociodemographic variables were computed.

**RESULTS:**

The patients’ mean age was 31.97±11.02 years. The mean hospitalization period was 18.08±15.25 days. The grade of burn was III in 75.7% patients. Among the acid-burn patients, 64.8% suffered from <20% of total body surface area burn. Most affected part of the body was Head /face/neck 17 (45.9%). Most of the acid-burn occurred from attack (67.6%). Burns mortality rate for this study was identified 8.1% (N=3).

**CONCLUSION:**

The results of this study showed high acid attacks rates. Prevention strategies must be coordinated at national level. So acid-burn patients have to receive the best medical care possible, first locally and then in a specialized center.

## INTRODUCTION

Burn injuries are one of the very important health problems that cause to prolonged hospitalization and hence increased expenses for patients, their families and society.^1^ Burn injury is most common in developing countries, especially in poor socioeconomic and rural areas.^2^ Between 2.4% until 10.7% of burn injuries across world are due to chemical exposure.^3^ In the past years, an increase has been detected in using chemical agents in aggressions involving domestic violence, especially to women, splashing them on the face and body.^4^ Most of the chemical burn injuries are due to assault or accidental.^5^

An increasing occurrence of acid attacks observed in several countries, such as Iran^6^ is an alarm for medical, social, and government authorities to explore solutions to prevent such terrible acts from happening and mitigate further complications to their victims.^7^ Researcher reported that Jamaica, Bangladesh, and Taiwan have the highest incidence of acid assaults.^8^ Burn injuries are the fourth most common type of trauma worldwide, after traffic accidents, falls and interpersonal violence.^9^

Burn is the third cause of mortality after accidents and drowning in the United States; and is the sixth largest cause of mortality in Iran.^10^ The epidemiology of burn injury is diverse across the world also within a country due to differences in the cultural and socioeconomic factors and the availability of health-care facilities.^11^ In this study, we present the epidemiology and outcome of acid-burns in a tertiary health care center in Tehran, Iran. Acid burn injuries, their severity, site involved, mortalities, their outcomes, and some other variables were evaluated.

## MATERIALS AND METHODS

This study was conducted in Motahari Referral Hospital, Tehran from 2005 to 2014. This Burns Unit is the only regional referral center for all burn injuries in the city. Motahari Burn and Reconstruction Centre is one of the few highly equipped tertiary burn centers in Iran, providing care to burned patients from the province of Tehran and to complicated cases referred from other centers across the Iran. We conducted a retrospective descriptive study in Motahari Hospital in Tehran, Iran. All of 37 acid-burn patients, during this 10 years, were calculated to identify the epidemiology of burns in this hospital. 

All patients with acid-burn injury were studied consecutively from the burn wards documents. No patient was excluded from the study. The total number of 37 burn admissions in a period of 10 years were subjected to a check list to obtain the following data: age, sex, brief description of the event, site affected the Total Burned Surface Area (TBSA) incurred, dates of admission and discharge. Outcome was recorded as patient survival or death. One trained research assistant, with the authors supervising, recorded data by use of a well-designed check list of the burns patients. The trained research assistant was nurse working in burn ward and the surgical ward with experience in burns management.

Other variables were specifically on what, what circumstance, how long was the hospital stay. The check lists were collected by reviewing medical documents at the burn unit of the hospital. All cases were selected for study but documents were excluded, if more than 20 percent of the required data were incomplete. The Statistical Package for the Social Sciences (SPSS, SPSS Inc., version 22.0, Chicago, IL, U.S.A.) was used for data analysis. Descriptive statistics (means with standard deviations or frequency distribution) of sociodemographic variables were computed. 

## RESULTS

Generally 37 acid-burn patients were evaluated. Male acid-burn patients almost were more than female patients (male=19 and female=18). The male to female ratio of the acid-burn patients was 1.06:1. The patients’ mean age was 31.97±11.02 years (range, 7 to 52 years old). Elderly age group was affected most (48.6% in ˃30 year’s age group). Married subjects were more affected (54.1% vs 45.9%). The mean hospitalization period was 18.08±15.25 (1-75) days. The majority of the acid-burn patients spent more than 21 days in the hospital (32.4%).

The grade of burn was III in 75.7% and II in 24.3% of patients. With regards to the severity of the acid-burns, most of the patients were deep burns which required many days of admission before the patients could be discharged from the hospital to attend surgical out patients’ services. Among the acid-burn patients, 29.7% suffered from <10% of total body surface area burn, 35.1% from 11-20% and the rest (35.1%) >20% of total body surface area burn. The body surface burn percentage mean was 17.16±1.75. Most affected part of the body was head/face/neck (N=17, 45.9%), next was trunk and hands (N=14, 37.8%). As most parts of acid-burn patient’s body affected by acid-burn, so the frequency was more than 37 patients (Table 1). 

**Table 1:** Distribution of the acid-burn patients (n=37) by socio-demographic characteristics, hospital stay, grade of burn, total body surface area (TB1`SA) burn, and parts of the body

**Variables **	**Frequency**	**Percentage**
Age		
˂10	1	2.7
11-20	2	5.4
21-30	16	43.2
˃30	18	48.6
Sex		
Male	19	1
Female	18	48.6
Marital status		
Single	17	45.9
Married	20	54.1
The duration of the hospital stay		
˂1 day	3	8.1
2-3 days	3	8.1
3-5 days	3	8.1
1 week	0	0
2 weeks	8	21.6
3 weeks	8	21.6
>3 weeks	12	32.4
Grade		
I	0	0
II	9	24.3
III	28	75.7
% of TBSA burn		
1-10%	11	29.7
11-20%	13	35.1
21-30%	8	21.6
31-40%	4	10.8
41-50%	1	2.7
Area		
Head /face/neck	17	45.9
Trunk Hands	14 14	37.8 37.8
Feet	7	18.9
Wrist and Ankle	12	32.4
Multiple organs	13	35.1

The results indicated that violence acid-burn injury was the most common mode of injury among patients. Accidents as the cause of acid-burn injury were responsible for burns in 7 (58.3%) and 5 (41.7%) of male and female patients, respectively, while these figures for singles and married cases were 3 (25%) and 9 (75%), respectively. Regarding violence as acid burn reasons were more in females (N=13, 52%) than males (N=12, 48%). These figures were 14 (56%) and 11 (44%) for singles and married patients, respectively.

Frequency of acid burns in survived patients was 91.9% (N=34) and dead ones was 8.1% (N=3). Regarding mean age, hospital stay, and %TBSA of the acid-burn patients regarding survived subjects was 31.94±10.98, 16.85±14.87 and 16.17±5.48 and in dead cases, the figures were 32.33±14.04, 32.00±14.93 and 28.33±12.2, respectively.

The frequency of burn degree in Table 2 indicates that, the highest frequency of burn degree, and mortality was related to ˃30 age group. Also results indicated that the highest mean±SD hospital stay was related to 11-20 age group. The results showed that acid-burn married women (N=12, 66.7%) were more than acid-burn married male patients (N=8, 94.75). These figures in males was inverse as 57.9% (N=11) in males and 33.3% (n=6) in female burn patients. Mean age in male cases was 33.47±10.72 and for female cases was 30.38±11.43. The survival rate was 94.7% (N=18) in males and 88.9% (N=16) in females. The figures for %TBSA in male acid burn patients were 14.47±11.56 in male cases and 20.00±16.49 in female subjects. 

**Table 2:** Distribution of the acid-burn patients by age groups, hospital stay, degree of burn, sex, and outcome

**Age groups **	**Mean±SD hospital stay**	**Degree of burn**			**Sex**		**Outcome**	
		**I**	**II**	**III**	**Male**	**Female**	**Survived**	**Dead**
˂10	5.00±1.02	0	0	1	0	1	1	0
11-20	30.50±13.43	0	0	2	0	2	1	1
21-30	17.75±9.02	0	3	13	10	6	16	0
˃30	17.72±19.54	0	6	12	8	9	16	2
Total	18.08±15.25	0	11	26	18	18	34	3

## DISCUSSION

Acid burn constitutes nearly 15% of total burn injuries.^12^ The present study revealed 37 acid burn patients, so a large number (N=19) of cases were men, although majority of acid burn occurred from attack. Among 37 acid-burn patients (N=18, 48.6%) were female and 19 (51.4%) were male. Study conducted by Islam and *et al.* showed 58.4% were female and 41.96% were male.^12^ Pande and *et al. * had 111 male and 100 female patients in their study.^13^ During Mohhamaddi *et al.*’s study, a total number of 135 severely burn patients, 93 were males (68.9%).^14^

In all these studies, males were found to be more affected than females. But in other similar studies in Iran and India, researcher found that male acid-burn were more than female acid- burn.^6^^,^^11^^,^^15^^,^^16^ Kitara *et al*. in their study had 53% females and 47% males.^17^ The difference in these studies may be due to different areas and reasons behind acid burn. Therefore, the occupational roles of the females in the family activities exposed them to more acid-burn injuries, in other hand may be men are at more risk of work-related burn injuries, because of these reasons we did not investigate this aspect in our study.

In the current study, patients’ mean age was 31.97±11.02 years. The majority of patients, in our study were ˃30 age group. This finding is not similar to those that showed the majority of patient being 10 years and younger.^17^ Asaria* et al.* found that 73.3% of patients had falling between the ages of 20 and 39 years.^18^ In other study, average age of the included patients was 38.04±11.91 years old.^19^ The average age of patients, study by pande *et al.* was 19.6±20.9.^13^ The patients’ mean age, in study conducted by Hashemi *et al.* was 36 years.^6^ The mean±SD age of the patients were 33±19.5, in Mohammaddi *et al.*^14^ This difference may be due to different sample size and which may be attributed to the particular institution’s admission policies, population under study, etc. 

In our study, out of 37 acid-burn patients, 64.8% had less than 20% of TBSA burned and rest of acid-burn patients had more than 20% of TBSA burn. These findings were consistent to some previous similar studies. Islam *et al.* found that 85.8% of participants had less than 20% of TBSA burns.^12^ Also, Kitara *et al.* showed that TBSA was as follows: <10% (21.7%), 10-14% (43.5%), 15-19% (21.7%) and 20-24 (13.0%).^17^ The majority of the patients sustained injuries with less than 20% of the total body surface area.

Our study showed that the duration of hospital stay ranged from 1-75 days with mean 18.08±15.25 days. Mohammadi *et al.* found that the duration of hospital stay ranged from 4-61 days with mean 20.2±18.0 days.^14^ Hashemi *et al.* showed that the mean hospitalization period, in their study was 39 days.^6^ These results showed, some studies reported higher or lower mean values, which may be due to this fact that their patient’s percentage TBSA, were more than our study. Our findings showed that sites most commonly affected included the face/ head/ neck (45.9%). 

Also Asaria *et al.* conducted that the face (86.7%), head and neck (66.7%) were most affected by acid-burn.^18^ In other previous studies, the face (78.0%), neck (51.5%) were the most frequently affected body areas.^19^^,^^20^ Head and neck region was found to be the most common part of body of injury accounting for 37% of the total injury.^15^ Facial involvement was most common and was observed in 72.8% of acid-burn cases.^6^ Therefore, most of one-third of acid-burn patients suffered visual impairment or complete blindness.^18^

In this study, 32.4% cases of acid-burn injury were accidental, and 67.6% cases were as criminal. Acid burn was mostly from violence in our country, but in other countries it is totally due to accidental or industrial hazards.^12^ A study from China reported 337 burn cases, of which 40 (10.5%) were due to criminal assault, and rest being related to industrial accidents.^21^ In East Azerbaijan, a province in Iran, 121 cases of chemical injury were reported that 111 cases (91.7%) were accidental, and 10 cases (8.3%) were as criminal assault.^22^ The frequency of this fact is increasing in developing countries,^7^ such as Iran, and in some industrial countries.^8^ While Vagardoost *et al.* evaluated epidemiology of burn in pregnancy in Iran, found that 28.9% of the burn cases were done with the intent to commit suicide.^23^

The common scenario of acid violence in majority third-world countries, and in some developed ones, is the victim’s spouse uses acid agent to gain revenge for an unsuccessful marriage or relationship provoked by social or economic reasons.^24^ A total of 37 patients, 67.6% in this study were cases of acid attacks; so 48.64% of them were females. In addition to the medical effects of acid burn, it has various social problems, especially in women, also this type of burn injury may making them dependent on other family members for their daily needs.^3^

The current study explored that mortality rate of acid-burn patients were 8.1. Some other studies also found that the mortality rate from acid assault was 2.0%^20^ and a total of three percent (3%) of deaths were recorded.^15^ Hashemi *et al.* found that five patients (8.4%) died owing to severe acid-burn injuries.^6^ The mortality rate is not high but the morbidity rate is more in case of acid burn and most of the patients occur from acid violence.^25^^-^^27^ Decreasing in mortality rates may be attributable to better diagnosis, management, and referral to specialized centers.

There were some limitations in this study, such as small sample size. The retrospective design meant that analysis could be done only for the data collected. It might have been appropriate and interesting to obtain data on the affected body part, and other variables such as socioeconomic status and educational level. Due to this was a retrospective study, some information, such as the mode of injury (suicide vs unintentional), was not detected which may have limited our ability to explain some of the findings. So we suggest future researches that be done by more information including mode of injury (suicide vs unintentional), where acid burn violence be done, socioeconomic status, educational status of victims. 

The results of this study showed high acid attacks rates (67.6%). Out of 37 acid-burn patients, 64.8% had less than 20% of TBSA burn, so mortality rate was less, and just 8.1% of patients died. Prevention strategies must be coordinated at national level. Acid-burn patients have to receive the best medical care possible, first locally and then in a specialized center.^25^ So an important factor involved in decreasing acid-burn morbidity and mortality among general population is planning educational programs and preventive measures.
